# Current application and future pathways of smart older adult care services in China

**DOI:** 10.3389/fdgth.2025.1656624

**Published:** 2025-10-31

**Authors:** Jie Xue

**Affiliations:** Department of Industrial Design, Tsinghua University, Beijing, China

**Keywords:** artificial intelligence, older adult care, health monitoring and management, medical service, emotional companionship

## Abstract

The aging problem in China has been more and more severe so that traditional older adult care service models are difficult to meet the growing demands of the older adult population. Smart older adult care, as an emerging service model, integrates artificial intelligence (AI) technologies to provide more personalized, convenient, and safe living services for the older adult. This paper analyzed the applications and development trends of AI in health monitoring and management, smart homes, emotional companionship, and medical services for older adult care and proposed some pathways to foster the healthy development of AI-enabled older adult care services by simplifying technological interfaces, enhancing user experience, strengthening data privacy protections, promoting industry standardization and so on.

## 1 Introduction

With the decline of birth rates and increase of life expectancy, a pronounced aging issue has emerged in China. According to the National Bureau of Statistics, the proportion of China's population aged 65 and above has been steadily increasing Since 2000 and is projected to reach approximately 30% by 2050. This phenomenon not only poses challenges to social and economic development but also creates unprecedented pressure on families and society. As the labor market changes and the younger population declines, traditional older adult care models face many kinds of challenges such as resource shortages, insufficient caregiving staff, and inconsistent service quality. It has become an urgent issue to effectively address the needs of the older adult and improve their quality of life.

As a new kind of technology, artificial intelligence (AI) can simulate and realize human intelligence to perform perception, reasoning, learning, decision-making, etc. AI technology is composed of machine learning, deep learning, natural language processing, computer vision, and so on. Machine learning can enable machines to learn and make predictions from experience through data training algorithms, while Deep learning can utilize multi-layer neural networks to process complex data so as to achieve significant results particularly in image recognition, speech recognition, and so on. Natural language processing enables computers to comprehend, generate, and manipulate human language, which has been applied in voice assistants and machine translation. In recent years, AI technologies have achieved a series of major breakthroughs in many kinds of areas, including human-computer interaction, image recognition, autonomous driving, smart healthcare, industrial automation, and so on. For instance, the large language models like GPT and BERT have enabled machines to comprehend and generate natural language text, facilitating more natural conversations, text processing. The e-commerce platforms such as Netflix and YouTube have enhanced the accuracy of personalized recommendation system by using Deep learning techniques. Computer Vision is a branch of artificial intelligence that enables computers to acquire knowledge and take actions from visual data through algorithms and technologies, which target is to allow computers to see and understand visual information like humans.

AI possesses robust capabilities in data acquisition, analysis, prediction, learning, and decision-making, making it highly applicable in smart older adult care services. It effectively enhances the quality of life for older adults while alleviating the burden on caregivers (1). Currently, in the field of older adult care services, AI technology is being applied across various aspects such as health monitoring and management, smart home systems, daily life assistance, emotional companionship, and medical services (2, 3). This paper provides a systematic study of the applications and development trends of AI in older adult care services in China, along with an in-depth analysis of challenges related to technical accuracy, user acceptance, and data privacy. Finally, a series of suggestions are proposed to promote the healthy development of AI technology in the field of older adult care services.

## 2 Applications of AI in smart older adult care in China

In the past few years, the developed countries have accumulated a great of experience and technology in the field of smart older adult care. They have invented some smart older adult care systems, including the use of sensor technology for real-time monitoring of older adult health data and developing personalized health management plans through data analysis (4). For instance, several technology companies in USA, have successfully created smart home-based older adult care platforms that use smart home devices, sensors, and other technologies to help the seniors' daily lives. Meanwhile, continuous AI theory and technology have been applied in China's older adult care services. The AI-powered products and service systems used in china have enhanced their quality of life and reduced the management costs of older adult care.

**Figure 1:**
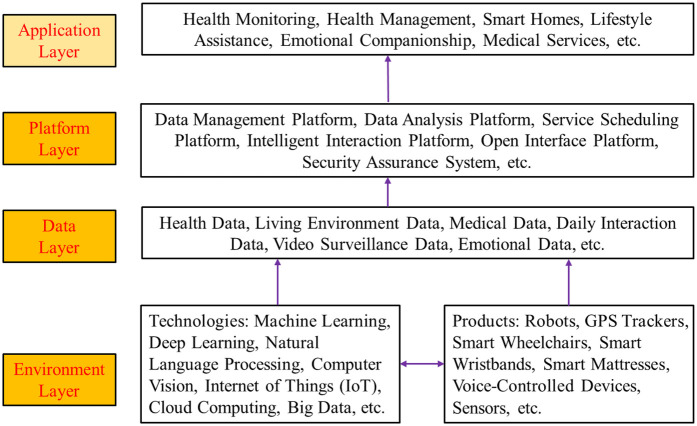
Application process flowchart of AI in older adult care services.

This paper analyzes the applications and development trends of AI in health monitoring and management, smart homes, emotional companionship, and medical services for older adult care and the challenges and strategies related to technological accuracy, user acceptance and data privacy in china. The AI application process flowchart for older adult care in china is shown in Figure 1. It can be seen that AI technologies have been applied in the areas of older adult care, including older adult health monitoring and management, smart home and living assistance, emotional companionship, and medical services in china.

### 2.1 Health monitoring and management

AI technologies have been intensively used in health monitoring and management in china, which is the fundamental component of smart older adult care services. In 2022, the market size of China's AI health monitoring and management reached 68.2 billion RMB, and it surpassed 85 billion RMB in 2023. It is projected that by 2025, the shipment volume of smart wearable devices in China will exceed 320 million units, with a market size of 42 billion RMB, in which smartwatches and health bands dominate the market, accounting for over 65% of the total share. AI can monitor seniors' physiological states (including heart rate, blood pressure, and blood glucose) and daily activities (such as physical activity and sleep quality) in real time by using wearable devices (such as smart wristbands, smartwatches, and smart badges), environmental sensors, and IoT technology (3). We can identify potential health issues in advance and issue warnings of the older adult using AI technologies, thereby take early intervention. For instance, Xiaomi smart wristbands can track real-time data such as ECG and blood oxygen levels, and detect abnormalities and alert caregivers or family members by analyzing these data using AI technologies. Similarly, Huawei smartwatches can offer high-precision blood pressure monitoring, analyze blood pressure trends through utilizing AI algorithms, and then provide health predictions and recommendations for the users. These health monitoring systems can significantly enhance safety of the older adult, particularly those with chronic conditions or limited mobility, by providing more efficient and accurate health management services.

Additionally, Xiang Yunhua and Wang Xiaohui investigated AI's supportive role in older adult health management in china, specifically in daily activity planning, sleep monitoring, and health interventions (5). However, they point out that the application of AI in the health management is still in its infancy, facing such practical problems as price barriers, information islands, insufficient cooperation among multi-stakeholders, and lack of professionals.

### 2.2 Smart homes

**Figure 2:**
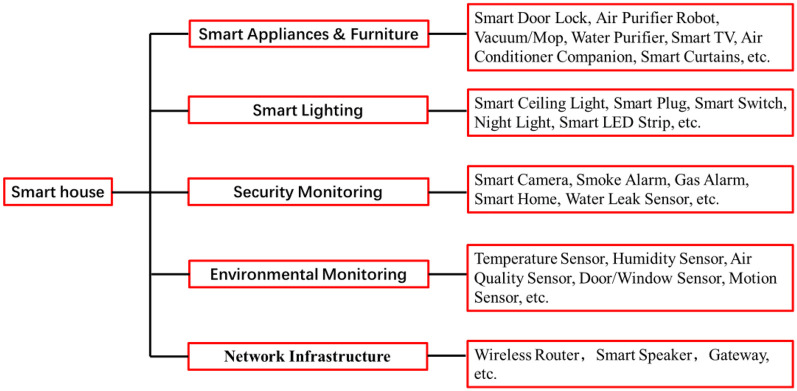
Smart home system.

Smart homes have garnered significant attention in China, increasingly playing a vital role in smart older adult care. A smart home primarily comprises five components: network infrastructure, environmental monitoring, security monitoring, smart lighting, and smart appliances and furniture, as illustrated in Figure 2. By utilizing smart home technology, we can provide convenient living services for the older adult. This technology has been identified as a key industry in China's 14th Five-Year Plan, which explicitly promotes the development of comprehensive home intelligence and digital households. According to authoritative forecasts, the smart home market in China is projected to reach 1.2 trillion RMB by 2025, with an annual compound growth rate exceeding 8% (6).

In recent years, numerous smart home devices have become integrated into the daily lives of older adult individuals (7). For instance, the Xiaomi Smart Speaker, which is equipped with the Xiao AI voice assistant, can control various smart devices through voice commands. This includes functionalities such as turning lights on and off, adjusting the temperature of air conditioners, and playing music. Additionally, it provides weather updates, news, and stock information, thereby enhancing convenience and entertainment. The Yeelight Smart Bulb can be managed via a mobile application or smart speaker, facilitating remote on/off control, brightness and color temperature adjustments, as well as scheduled operations and various lighting scenes, including reading mode, sleep mode, and party mode. The EZVIZ Smart Camera offers high-definition video surveillance, supporting remote viewing, two-way audio, motion detection, and human recognition, which ensures real-time home security. It can also integrate with other smart devices, such as automatically activating lights upon motion detection. The Kaadas Smart Lock features multiple unlocking methods, including 3D facial recognition and palm vein recognition. It additionally provides a remote peephole function, allowing users to check outside via their mobile phone, along with anti-tamper alarms and alerts for erroneous attempts to enhance security. The Haier Smart Refrigerator utilizes IoT technology for remote control through a mobile application, enabling temperature adjustments, checking food expiration dates, and creating management plans. Some models also include smart freshness preservation and sterilization functions. The Midea Smart Washing Machine facilitates remote operation through a mobile application, automatically selecting the optimal washing mode based on the type of fabric and the weight of the load. Additionally, it provides smart scheduling and fault diagnosis features, enhancing the convenience and efficiency of the laundry process. The DooYa V5 Series Smart Curtain Motor operates quietly and supports various control methods, including remote control, mobile application, and voice commands. This device enables precise automatic opening, closing, and positioning of curtains, as well as integration with other smart devices based on specific scenes. The Bull Smart Plug is characterized by its reliable quality and high safety standards, allowing for remote control through a mobile application. It supports scheduled on/off operations for appliances such as kettles, humidifiers, and fans, thereby facilitating smart management. In summary, a smart home environment not only enhances the quality of life for the older adult but also significantly reduces the workload for caregivers (8).

### 2.3 Emotional companionship and psychological support

Loneliness and depression are prevalent psychological health issues among the older adult. According to a survey conducted by the Tencent Research Institute, 98% of respondents consider utilizing AI companionship to address unmet social needs in their daily lives, with the potential demand for companion robots among the older adult estimated to reach approximately 420 billion RMB. On June 11, 2025, Shanghai Huihua Intelligent Technology Co., Ltd. launched China's first AI companion robot, named Xiao Bai, which features active dialogue and personalized interaction capabilities, as showcased at the Shanghai Senior Expo. AI technology can provide emotional companionship through virtual characters or chatbots, thereby alleviating feelings of loneliness. By employing natural language processing (NLP) and emotion analysis technologies, AI can engage in daily conversations with older adult individuals, understand their psychological states, and offer appropriate interactions based on their emotional fluctuations. AI-based virtual companion robots can help the older adult maintain a positive mental state through activities such as conversations, games, and music. Furthermore, AI can recommend suitable activities or therapeutic solutions tailored to the emotional needs of the older adult.

### 2.4 Medical services

China has strategically placed AI at the forefront of its healthcare transformation, focusing on meeting the demands of the older adult. In 2023, the Ministry of Industry and Information Technology introduced the innovative Robot Plus Application Action Plan, which methodically incorporates robotic technologies into geriatric care and clinical settings. The medical services powered by AI now cover from preventive health monitoring to advanced diagnostic assistance and virtual medical consultations. For example, Ant Group's state-of-the-art AI health companion, AQ, has formed partnerships with over 5,000 healthcare facilities and around one million healthcare professionals across the country, which provides a range of services, including health literacy initiatives, remote consultations, and analysis of diagnostic reports. Simultaneously, iFlytek has achieved notable progress with its Spark Medical Model V2.5 International Edition and the upgraded Xiaoyi app, which boasts over 24 million installations and has enabled more than 140 million AI-assisted medical interactions. Winning Health has embedded its cutting-edge WiNGPT technology into the WiNEX Copilot system, which enhances hospital operations and aids physicians in making diagnostic decisions.

## 3 Challenges and future direction of AI in smart older adult care

AI has significant potential in older adult care services, which can improve the life quality of the older adult, enhance care efficiency, and reduce the burden on caregivers. However, the adoption and application of AI in older adult care still face a series of challenges. This chapter will systematically analyze these challenges in smart older adult care and explore future development paths.

### 3.1 Technological maturity and reliability

While the application of AI in the field of older adult care is advancing, it should be noted that the current technological maturity still has deficiencies, especially in key areas such as health monitoring, disease prediction, and behavioral analysis of the older adult. For example, smart devices may not effectively identify sudden health crises such as cardiac arrest or stroke, which may lead to delayed or inaccurate emergency responses. In addition, the reliability of AI systems urgently needs to be improved. As noted, the older adult care services require continuous operation, but problems such as system failures, maintenance challenges, or network interruptions may disrupt services, thereby posing risks to the older adult. Therefore, a major focus should be on improving the reliability and safety of AI systems in the future (9, 10).

### 3.2 User acceptance and adaptability

The older adult always shows lower acceptance level and adaptability to new technology so that they may resist or possess apprehensions about smart devices (11–14). Besides, the effectiveness of AI-enhanced smart services can be compromised by the established habits and physical limitations of the older adult. For example, impairments in vision, hearing, or mobility might prevent them from using these devices efficiently, subsequently diminishing their overall functionality. Consequently, it is crucial to encourage digital literacy training for the older adult and to create straightforward, intuitive, and user-friendly interfaces to improve their interaction with smart devices.

### 3.3 Data privacy and security

The use of smart older adult care services requires gathering a significant amount of personal information, including health conditions, daily routines, and family details. The breaches of data or its improper use can result in serious repercussions, especially for older individuals who might be more vulnerable to fraud or cyber threats. Therefore, it is a crucial challenge to find a middle ground between collecting data and ensuring privacy (15).

### 3.4 Ethical considerations

Although AI can greatly improve the efficiency of the older adult care services, the older adult continues to need emotional support and personal interaction. Excessive dependence on AI technology might worsen isolation feelings of the older adult and negatively impact their mental wellbeing (16–19). Thus, it is an essential issue to find a balance between technological support and personal care.

### 3.5 Cost

The smart older adult care services significantly depend on high-quality equipment, including smart home gadgets, health monitoring tools, and robotic systems, together with advanced software applications so that the continual great costs might obstruct their accessibility. To address this challenge, it is crucial to create industry standardizations, merge medical resources, encourage collaboration across different sectors, and introduce policy incentives designed to lower costs and promote the integration of AI within the older adult care industry in China.

## 4 Conclusion

The smart older adult care, including health surveillance, tailored assistance, intelligent living spaces, and emotional support, can deliver great aid to the older adult. As technology continues to evolve, the smart care services will become an essential element of the lives of the older adult. However, the smart older adult care services face various obstacles, including technology adaptability & acceptance, data privacy and security, ethical considerations, cost, and so on. Therefore, it is essential to enhance their dependability and safety, simplify technical interfaces, strengthen data privacy measures, address ethical issues, and reduce costs, which are crucial for the advancement of smart older adult care industry in China.

## References

[1] Wang Y Xie C Liang CY Zhou PY Lu LY. Association of artificial intelligence use and the retention of older adult caregivers: a cross-sectional study based on empowerment theory. J Nurs Manag. (2022) 30:3827–37. 10.1111/jonm.13823 36177709

[2] Wang YH Zeng HY Lv FL Wang JC. Analysis of demand and influencing factors for smart senior care among older adults in underdeveloped regions of western China: a case study of Lanzhou. Front Public Health. (2024) 12:1337584. 10.3389/fpubh.2024.1337584 38939563 PMC11210194

[3] Fu LP Pei TP Yang J Han JR. How smart senior care can achieve value co-creation: evidence from China. Front Public Health. (2022) 10:973439. 10.3389/fpubh.2022.973439 36211655 PMC9533210

[4] Alberto GJ María LPL Juan A GP Diego RP Gómez-Pulido JM. Predictive health monitoring: leveraging artificial intelligence for early detection of infectious diseases in nursing home residents through discontinuous vital signs analysis. Comput Biol Med. (2024) 174:108469. 10.1016/j.compbiomed.2024.108469 38636331

[5] Xiang YH Wang XH. Research on older adult health management in the era of artificial intelligence. J Xinjiang Norm Univ. (2019) 40(04):98–107. 10.14100/j.cnki.65-1039/g4.20190507.003

[6] Zhongyan Puhua Industry Research Institute. 2025 China smart home industry market analysis research report: in-depth analysis (2025). Available online at: https://m.chinairn.com/scfx/20250326/182016292.shtml (Accessed March 26, 2025).

[7] Extraordinary Partners. China's first AI active companion robot was officially released at the Elderly Care Expo (2025). Available online at: https://www.toutiao.com/article/7508978172734587427/?upstream_biz=doubao&source=m_redirect&wid=1761545917354 (Accessed May 27, 2025).

[8] Zhang T. AI has become a new force in serving an aging society (2025). Available online at: https://www.mca.gov.cn/n152/n166/c1662004999980004517/content.html (Accessed April 16, 2025).

[9] Ding YZ. Preliminary exploration of high-quality development technical solutions for older adult care services under the perspective of AGI. Soc Secur Rev. (2024) 8(03):90–107.

[10] Qu YT. Research on issues and countermeasures of smart older adult care (Doctoral dissertation). Hebei Normal University, Shijiazhuang, China. (2023).

[11] Wang QX Liu JN Zheng Y. Evolutionary game analysis of community older adult care service regulation in the context of “internet +”. Front Public Health. (2022) 10:1093451. 10.3389/fpubh.2022.1093451 36620239 PMC9815532

[12] Chen JL. Research on community smart older adult care service models in the context of internet+. Intell Comput Appl. (2020) 10(02):308–310+16.

[13] Tang KY Liang HJ. Modernization and improvement path of smart older adult care capabilities. Soc Sci Front. (2022) 02:230–6.

[14] Zhou WS XiY L Liu H. Research progress and prospects on empowering older adult care services with digital technology. Soc Secur Res. (2024) 01:100–11.

[15] Geng YZ Wei YN Zhou J. Investigation into the development issues of “internet+ older adult care services”. Macroecon Manag. (2019) 01:71–7. 10.19709/j.cnki.11-3199/f.2019.01.013

[16] Zhao N Liu SL Sun XN. Application and ethical considerations of artificial intelligence in home older adult care. Chin Med Ethics. (2021) 34(12):1590–4. 10.12026/j.issn.1001-8565.2021.12.13

[17] Tong F. Construction and application strategies of multi-system interaction smart older adult care service system. J Nantong Univ. (2021) 37(02):89–96.

[18] He M Hou RY Yin M. Reflections on the ethical dilemmas of artificial intelligence in older adult care. Med Philos. (2024) 45(06):27–30+41.

[19] Zhao Y. Ethical dimensions of intelligent older adult care. J Shanghai Jiao Tong Univ. (2022) 30(01):63–70. 10.13806/j.cnki.issn1008-7095.2022.01.007

